# Detection of bulk feed volume based on binocular stereo vision

**DOI:** 10.1038/s41598-022-13075-7

**Published:** 2022-06-04

**Authors:** Zhihai Liu, Zhenrui Dai, Qingliang Zeng, Jinxia Liu, Feiyi Liu, Qing Lu

**Affiliations:** 1 College of Transportation, Shandong University of Science and Technology, Qingdao, 266590 China; 2 College of Mechanical and Electronic Engineering, Shandong University of Science and Technology, Qingdao, 266590 China; 3 College of Information Science and Engineering, Shandong Normal University, Jinan, 250358 China; 4 College of Automation, Shandong University of Science and Technology, Qingdao, 266590 China

**Keywords:** Computational science, Computer science

## Abstract

The volume detection of medical mice feed is crucial to understand the food intake requirements of mice at different growth stages and to grasp their growth, development, and health status. Aiming at the problem of volume calculation in the way of feed bulk in mice, a method for detecting the bulk volume of feed in mice based on binocular stereo vision was proposed. Firstly, the three-dimensional point coordinates of the feed's surface were calculated using the binocular stereo vision three-dimensional reconstruction technology. The coordinates of these dense points formed a point cloud, and then the projection method was used to calculate the volume of the point cloud; and finally, the volume of the mice feed was obtained. We use the stereo matching data set provided by the Middlebury evaluation platform to conduct experimental verification. The results show that our method effectively improves the matching degree of stereo matching and makes the three-dimensional point coordinates of the obtained feed's surface more accurate. The point cloud is then denoised and Delaunay triangulated, and the volume of the tetrahedron obtained after the triangulation is calculated and summed to obtain the total volume. We used different sizes of wood instead of feed for multiple volume calculations, and the average error between the calculated volume and the real volume was 7.12%. The experimental results show that the volume of the remaining feed of mice can be calculated by binocular stereo vision.

## Introduction

Mice are the first choice of experimental animals in many fields such as teaching, scientific research, and experimental research. The detection of the feeding amount of experimental mice is an important prerequisite to ensure their healthy growth and accurate experimental results. At present, most of the testing of the feed consumption of laboratory mice adopts the manual weighing method, which is labor-intensive, easy to fatigue, and easy to cause residual feed contamination. In addition, it can also be achieved by measuring the volume for conversion; the volume calculation problem can be seen as an extension of the problem of measuring the surface shape or depth of the animal feed. The volume calculation can be done by estimating the space occupied by the point cloud model obtained by 3D reconstruction with the help of machine vision, image processing, and other technologies.

The research algorithms for obtaining physical model volume based on a three-dimensional point cloud can be roughly divided into the following four categories: (1) Convex hull algorithm^1,2^ The convex hull model is used to approximate the irregular object, and the convex hull model is divided and accumulated or decomposed into two triangular mesh surfaces, and the corresponding projection volume is calculated by the orthographic projection method, and the difference is the desired object volume. This method is suitable for convex models, and the calculation error of non-convex models is large. (2) Model reconstruction method^3^. The physical model of the point cloud is constructed by triangular patches to obtain the volume of the object. This method is greatly affected by the point cloud density, the number of generated triangular meshes, and the pointing accuracy, and it is easy to generate holes. (3) Slice method^4,5^. The point cloud is sliced along the direction perpendicular to the coordinate axis, and the total volume is obtained by accumulating slice volumes. This method is affected by the slice thickness. The smaller the slice thickness, the higher the computational accuracy but the decrease in computational efficiency. (4) Projection method11. First, the point cloud projection is triangulated, and then the projection point and its original corresponding point are constructed to form a pentahedron, and the total volume is obtained by accumulating the volume of the pentahedron. The algorithm is also prone to holes. Volume measurement by point cloud has a vital application basis in the fields of coal^6^, trees^7,8^, and hospital disease diagnosis^9,10^.

This paper uses binocular stereo vision technology to reconstruct the object to obtain the point cloud model. The advantages of this technology are that the measurement speed is fast, the measurement accuracy is high, and high-density point cloud data can be obtained. Binocular stereo vision is a technology that uses sensors to simulate the human eye to collect images and process images, which can map three-dimensional spatial information into two-dimensional images. The binocular stereo vision technology includes five steps: image acquisition, camera calibration, image correction, stereo matching, and three-dimensional reconstruction. Stereo matching is the most critical step in this process. Scharstein and Szeliski^11^ summarized stereo matching into four processes: matching cost computation, cost aggregation, disparity calculation, and disparity refinement. Stereo matching algorithms can be divided into local, global, semi-global, and deep learning-based stereo matching algorithms according to their characteristics.

The local stereo matching algorithm is also called the region-based or window-based image matching method. This method takes the pixels in the local window of the pixels to be matched as constraints and calculates all the pixels in each disparity window by moving the window horizontally in another perspective. And then, using the Winner-Takes-All(WTA) principle, the minimum matching cost value is selected as the point disparity. Hamzah et al.^12^ introduced the Sum of Gradient (SG) matching algorithm, using the amplitude difference of fixed window size for matching, an Adaptive Support Weight (ASW) algorithm based on iterative Guided Filter (GF) is proposed, which reduces the influence of noise and preserves the edges of objects. Kitagaw et al.^13^ proposed a guided filter matching method based on the response of the Different of Gaussian (DoG). The matching cost is calculated using the Sum of Absolute intensity Differences (SAD) of the grayscale difference and the weighted sum of the gradient values of adjacent pixels. An appropriate window is selected according to each pixel, and the cost matrix is smoothed and aggregated by guided filtering.

The global stereo matching algorithm continuously optimizes the allocated disparity value by constructing a global cost function until the energy of two terms in the objective function is minimized. That is, the data term penalizes the solution inconsistent with the target data, and the smoothing item forces the piecewise smoothing assumption for adjacent pixels. The global method can produce better results, but the computational cost is high, and it is not suitable for real-time systems. The global stereo matching algorithm usually includes three steps of matching cost computation, disparity calculation, and disparity optimization, and no cost aggregation is performed. Zhou et al.^14^ proposed a dynamic programming stereo matching algorithm based on differential equations, using all matching cost functions, smooth energy functions, and occlusion cost functions. The diffusion speed of the smooth energy function includes gradient information, line points, and corner points. The matching degree of the depth discontinuity area is improved, and the error rate of the disparity is reduced. Hong and Chen^15^ transformed the stereo matching problem into the energy minimization problem in the segmentation domain and used the graph cut technique to quickly approach the optimal solution, assigning the corresponding disparity plane to each segmentation. The algorithm has a high matching degree between the discontinuous disparity boundary and the occluded part in the textureless area.

The Semi-Global Matching (SGM) algorithm was proposed by Hirschmüller^16^, which also adopts the idea of optimizing the energy function, and estimates the optimal disparity value by minimizing the global energy function. Different from the global matching method, this algorithm transforms the optimization problem of a two-dimensional image into a one-dimensional optimization problem of multiple paths, calculates the matching cost of pixel points, aggregates the path cost from 8 or 16 directions, and uses the WTA method to calculate disparity. Wan et al.^17^ proposed an SGM method combining matching costs for the problem of low matching accuracy of existing stereo matching algorithms for weak texture and discontinuous disparity regions. The matching cost is calculated using a combination of adaptive weight SAD and Absolute intensity Differences of Grads (GAD), and an 8-path semi-global method is used for cost aggregation.

With the rapid development of deep learning, it has become an important method to study the field of binocular stereo vision, and the stereo matching method based on deep learning improves the performance of stereo vision applications. Zbontar and Lecun^18^ proposed a method to obtain depth information from rectified image pairs, first using Convolutional Neural Networks (CNN) for matching cost computation, then through cross cost aggregation and Semi-Global Matching refinement, and finally using the left and right consistency constraints to eliminate errors in the occluded area. Mayer et al.^19^ first applied the end-to-end network to the stereo matching algorithm. By training the end-to-end convolutional neural network, using the encoder-decoder structure, the correlation quantity was calculated from the left and right image features, and the disparity map was directly output. Chang and Chen^20^ proposed a pyramid stereo matching network PSMNet composed of two modules of spatial pyramid pooling and a three-dimensional convolutional neural network. The capacity of the global context information is exploited by the spatial pyramid pooling module to aggregate contexts of different scales and locations into a cost volume, and then the three-dimensional CNN uses stacked multiple hourglass networks combined with intermediate supervision to learn the regularized cost volume.

The rest of this article is organized as follows. “Binocular stereo vision geometric model” introduces the geometric model of binocular vision, including the relationship between the four coordinate systems and the principle of the binocular vision model. Section “Stereo matching” proposes a cost computation method for weighted fusion of gradient, Squared Difference (SD), and Census Transforms and introduces other steps of stereo matching. Section “Volume computation” introduces the volume calculation method of the projection method based on Delaunay triangulation. Section “Experimental results and analysis” conducts experiments on stereo matching and volume calculation and analyzes the experimental results. Section “Conclusion” concludes the article.

## Binocular stereo vision geometric model

**Figure 1:**
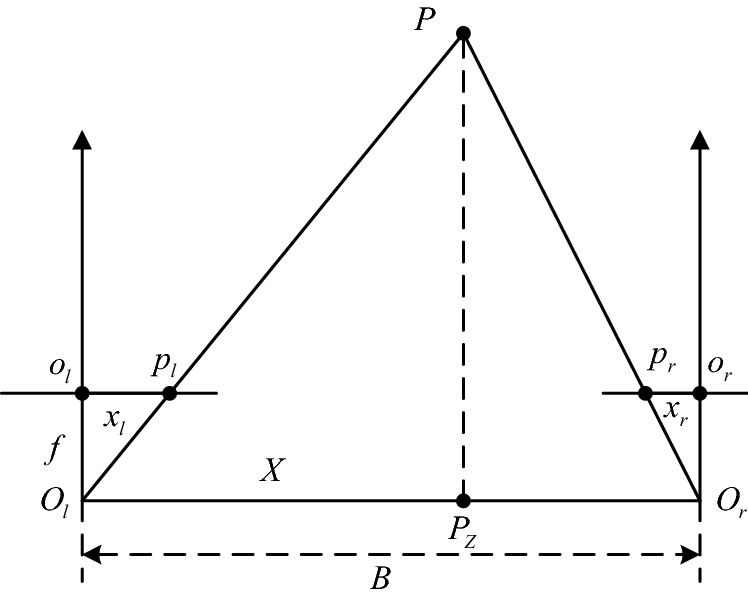
Standard stereos vision system^21^.

The binocular cameras are located at the same height, and the imaging plane is on the same horizontal plane. When shooting the same object, the angle formed between the left camera and the right camera and the observation object is different, and there will be a position difference on the imaging plane of the camera-this position difference is also known as disparity. As shown in Fig. 1, the coordinates of a point *P* in the space point in the imaging plane of the left camera and the right camera are $p_{l} (x_{l} ,y_{l} )$ and $p_{r} (x_{r} ,y_{r} )$, at this time $y_{l} = y_{r}$, then the disparity of this point is $d_{0}$. According to the geometric information, the expressions of $x_{l}$ and $x_{r}$ can be obtained, as shown in Eq. (1):

1 $$ \left\{ {\begin{array}{*{20}l} {x_{l} = f \cdot \frac{X}{Z}} \hfill \\ {x_{r} = f \cdot \frac{X - B}{Z}} \hfill \\ \end{array} } \right. $$

where *B* is the camera baseline distance, *f* is the focal length of the camera, and *Z* is the depth information of the spatial point *P*. Then, the calculation equation of disparity $d_{0}$ is shown in Eq. (2).

2 $$ d_{0} = x_{l} - x_{r} = \frac{B \cdot f}{Z} $$

The three-dimensional coordinates of the space point *P* can be calculated from Eqs. (5) and (6), as shown in Eq. (3). ^22^:

3 $$ \left\{ {\begin{array}{*{20}c} {X = \frac{{B \cdot x_{l} }}{{d_{0} }}} \\ {Y = \frac{{B \cdot y_{l} }}{{d_{0} }}} \\ {Z = \frac{B \cdot f}{{d_{0} }}} \\ \end{array} } \right. $$

Assume that the coordinates of $p_{l}$ and $p_{r}$ in the image pixel coordinate system are $p_{l} = (u_{l} ,v_{l} )$ and $p_{r} = (u_{r} ,v_{r} )$, then the disparity $d$ of $p_{l}$ and $p_{r}$ in the image pixel coordinate system can be expressed as $d = u_{l} - u_{r} { = }d_{0} /d_{x}$. According to the inverse relationship of parallax $d$, $d_{0}$ and Eq. (3), the three-dimensional coordinates of space point $P$ in the image pixel coordinate system can be obtained:

4 $$ \left\{ {\begin{array}{*{20}l} {X = \frac{{B \cdot (u_{l} - u_{0} )}}{d}} \hfill \\ {Y = \frac{{B \cdot f_{x} \cdot (v_{l} - v_{0} )}}{{d \cdot f_{y} }}} \hfill \\ {Z = \frac{{B \cdot f_{x} }}{d}} \hfill \\ \end{array} } \right. $$

## Stereo matching

The core of stereo matching is to find the corresponding pixels in the left and right images to calculate the disparity and then obtain the depth information of the object. Image noise, lighting, and texture all affect the matching results. Designing a corresponding stereo matching method for a specific environment is of great significance to obtain an accurate disparity map.

### Gradient-SD-census matching cost computation

The matching cost is a measure of the similarity of the corresponding pixels between the left and right images. Generally, the pixel with the highest similarity within the parallax search range of the left and right images is selected as the matching point through the similarity measure function. The Census transform is a local non-parametric transform that transforms the relative size relationship between pixels and surrounding pixels on grayscale into a binary string. For any pixel *p* in the image *I*, select a rectangular transformation window 1 centered on *p*. Then compare the gray level of other pixels in the rectangular transformation window with p; if the gray level of the adjacent pixel is greater than p, it is recorded as 1; otherwise, it is recorded as 0. Finally, these comparison results are concatenated into a binary string. As shown in Eq. (5):

5 $$ T_{I} (p) = \mathop \otimes \limits_{{q \in \omega_{p} }} \xi (I(p),I(q)) $$

where $\otimes$ represents the binary string concatenation operation, $I(p)$ and $I(q)$ are the grayscale values of pixel *p* and pixel *q* respectively, $\zeta (I(p),I(q))$ is a binary function, and its definition is shown in (6):

6 $$ \zeta (I(p),I(q)) = \left\{ {\begin{array}{*{20}c} {0 \, I(p) \ge I(q) \, p,q \in \omega_{p} } \\ {1 \, I(p) < I(q) \, p,q \in \omega_{p} } \\ \end{array} } \right. $$

After completing the Census Transform, it is necessary to calculate the similarity of the two regions through the obtained bit string, and the similarity of the regions can be reflected by the size of the Hamming distance. The bitwise summation after bitwise XOR is the Hamming distance. The definition of Hamming distance is shown in Eq. (7), and the size of its value affects the accuracy of stereo matching.

7 $$ C(x,y,d) = Ham(p_{l} (x,y),p_{r} (x + d),y) $$

The traditional Census Transform mainly depends on the gray value in the window when calculating the initial cost and does not use the specific gray value of the image pixel for calculation. Therefore, the Census Transform has better robustness to noise and illumination, and it is relatively simple. However, the Census Transform has a strong dependence on the gray value of the center point pixel, and there is a problem of a high error rate in weak texture and edge regions.

In order to solve the problems of the above Census Transform, gradient information and SD are introduced, and the three methods are weighted and fused to calculate the cost. Gradient information is an attribute of the image itself, and it has good stability to the noise and illumination of the image, so the gradient information of the image can be used to measure the similarity of two different images. It can prevent the edge area from being too smooth and further improve the robustness of the matching algorithm to interference. The gradient information property of an image can be expressed as:

8 $$ C_{grad} (p,d) = \, |\nabla_{x} I_{l} (p) - \nabla_{x} I_{r} (p,d)| + |\nabla_{y} I_{l} (p) - \nabla_{y} I_{r} (p,d)| $$

where $C_{grad} (p,d)$ is the gradient-based matching cost of the pixel $p$ when the disparity value is $d$, $I_{l} (p)$ is the gray value of the pixel $p$ in the left image, $\nabla_{x}$ and $\nabla_{y}$ are the single-channel gradient operators in the $x$ and $y$ directions, respectively, $I_{r} (p,d)$ is the gray value corresponding to $p$ point in the right image and the disparity is $d$ pixel.

SD is the sum of squares obtained by taking the difference between a pixel and the pixels in its neighborhood. It has a good matching effect in the rich texture area of the image, but it is easily affected by light and noise, which can be expressed as:

9 $$ C_{SD} (p,d) = \frac{1}{3}\sum\limits_{i = R,G,B} {(I_{i}^{Left} (p) - I_{i}^{Right} (pd))}^{2} $$

In order to better fuse the Census Transform, SD, and gradient information, the method of weighted fusion of the three are adopted. Because the Census transform has better adaptability to changes in illumination, SD has better effects in weak texture areas and repeated texture areas, and gradient information constrains the smoothness of image information in the edge area. To get a more accurate matching effect, the specific equation is shown in (14):

10 $$ C(p,d) = \rho (C_{grad} (p,d),\lambda_{grad} ) + \rho (C_{SD} (p,d),\lambda_{SD} ) + \rho (C_{census} (p,d),\lambda_{C} ) $$

where $\lambda_{C}$, $\lambda_{SD}$, $\lambda_{G}$ represent the weight of Census Transform, SD, and gradient information, respectively, and 1 is the robust function of 1, which is defined as^23^:

11 $$ \rho (c,\lambda ) = 1 - \exp ( - \frac{c}{\lambda }) $$

Using the exponential form can better control the Census Transform, the values obtained by SD and gradient information, and will not affect the output results due to the excessive variation of one of the values. At the same time, parameter control is used in the equation to allow slight anomalies. The influence of the value ensures the accuracy and reliability of the algorithm and also improves the accuracy of the algorithm. The specific equation is shown in Eq. (12):

12 $$ C = 3 - \exp ( - \frac{{C_{grad} (p,d)}}{{\lambda_{G} }}) - \exp ( - \frac{{C_{SD} (p,d)}}{{\lambda_{SD} }}) - \exp ( - \frac{{C_{census} (p,d)}}{{\lambda_{C} }}) $$

### Cost aggregation

**Figure 2:**
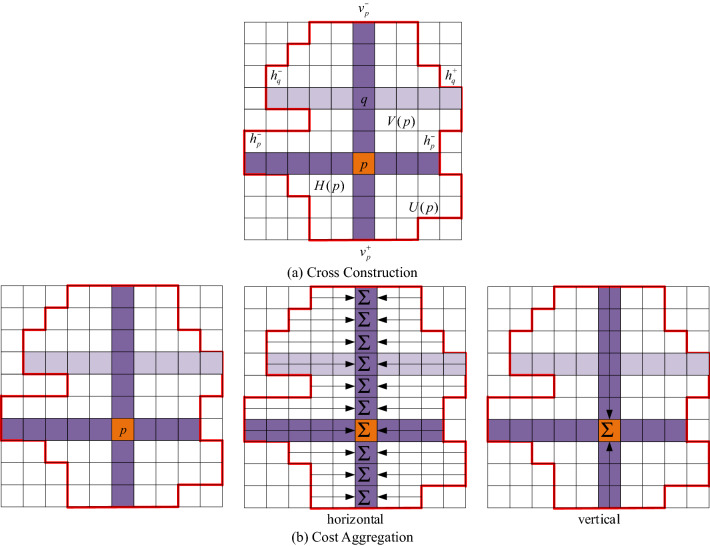
Cross-based cost aggregation.

Due to the low discriminative degree of the matching cost of a single pixel, the disparity obtained by the matching cost computation is not reliable. The cost aggregation can aggregate the matching cost of each pixel on the support domain, which can reduce the noise and blur caused by the matching of the initial matching cost. This paper focuses on the cost aggregation method based on cross-based by Zhang et al.^24^ and Mei et al.^25^. The intersection-based support field is first constructed by extending each pixel *p* horizontally and vertically to adjacent pixels with similar intensities to it, denoted as $V(p)$ and $H(p)$:

13 $$ \left\{ {\begin{array}{*{20}c} {V(p) = \{ (x,y)|x \in [x_{p} - l_{v}^{ - } ,x_{p} + l_{v}^{ + } ],y = y_{p} \} } \\ {H(p) = \{ (x,y)|y \in [y_{p} - l_{h}^{ - } ,y_{p} + l_{h}^{ + } ],x = x_{p} \} } \\ \end{array} } \right. $$

where $\{ l_{v}^{ - } ,l_{v}^{ + } ,l_{h}^{ - } ,l_{h}^{ + } \}$ is the length of the four arms, as shown in Fig. 2a, taking the left arm as an example, the limit conditions of the arm length are:

1. $D_{C} (p_{l} ,p) < \tau_{1} \, and \, D_{C} (p_{l} ,p_{l} + (1,0)) < \tau_{1}$,$D_{C} (p_{l} ,p)$ is the color difference between $p_{l}$ and $p$, $D_{C} (p_{l} ,p_{l} + (1,0))$ is the color difference between $p_{l}$ and a pixel $p_{l} + (1,0)$ in front of its arm, $\tau_{1}$ is the threshold set to avoid the extension of the arm across edge pixels. The definition of color difference is $D_{C} (p_{l} ,p) = \max_{{i{ = }R,G,B}} \left| {I_{i} (p_{l} ) - I_{i} (p)} \right|$.
2. $D_{S} (p_{l} ,p) < L_{1}$, $D_{S} (p_{l} ,p)$ is the length of the space between $p_{l}$ and $p$, and $L_{1}$ is the set threshold. The definition of space length is $D_{S} (p_{l} ,p) = \left| {p_{l} - p} \right|$.
3. $D_{C} (p_{l} ,p) < \tau_{2} \, , \, if \, L_{2} < D_{S} (p_{l} ,p) < L_{1}$,$\tau_{2}$ is the set threshold, $\tau_{2} < \tau_{1}$, $L_{2}$ is the set threshold, $L_{2} < L_{1}$. This limitation is that when in weakly textured areas, having longer arms without making them too long ensures that the arms extend in areas of similar color.

Then, build the support window with the cross arm as the skeleton. Simply expand horizontally as above for each point on the vertical arm. The process is shown in Fig. 2b. Denote the set of points on the horizontal arm and the vertical arm with $H(p)$ and $V(p)$ respectively, then the adaptive support area $U(p)$ of the pixel $p$ can be expressed as^24^:

14 $$ U(p) = \bigcup\limits_{q \in V(p)} {H(p)} $$

Pixel *q* is the neighborhood pixel of the pixel *p* in the vertical direction. This method uses not only the support area $U(p)$ of the pixel *p* but also the support area $U^{\prime}(p)$ of the corresponding pixel $q = (x,y - d)$, then the aggregate pixel cost between pixels *p* and *q* is defined as:

15 $$ C(p,d) = \frac{1}{{\left\| {U_{d} (p)} \right\|}}\sum\limits_{{q \in U_{d} (p)}} {C(q,d)} $$

where $U_{d} (p,d) = \{ (x,y)|(x,y) \in U(p),(x,y - d) \in U^{\prime}(p)\}$, $\left\| {U_{d} (p)} \right\|$ is the number of pixels in $U_{d} (p)$ for normalized aggregation cost $C(p,d)$.

### Disparity computation

The disparity computation is to calculate the optimal disparity value of each pixel through the cost matrix after cost aggregation. Generally, the local optimization method of WTA^26^ is used to calculate the initial disparity of each pixel from the aggregated cost volume $C^{A}$ pixel by pixel. Specifically, for each pixel *p*, the WTA optimization strategy selects the disparity with the smallest aggregation cost value as the optimal disparity $d_{p}$ of the pixel within the disparity range, namely:

16 $$ d_{p} { = }\mathop {\arg \min }\limits_{d \in D} C^{A} (p,d) $$

The disparity computation process generally takes the left view as the reference image, so the initial disparity map of the left view can be generated by mapping each pixel in the left view to its optimal disparity level according to the WTA optimization strategy.

### Disparity optimization

The initial disparity map obtained by disparity computation still has occlusions and mismatched pixels. In this section, various optimization methods are used to deal with disparity errors, mainly including outlier detection, proper interpolation, and sub-pixel enhancement.

**Outlier detection:** Using left–right consistency detection to find outliers in the disparity map. According to the uniqueness constraint of disparity, each pixel in the left image can find its disparity value in the right image correspondingly and judge whether the difference between the disparity of two pixels is less than the threshold of 1 pixel. If so, then the pixel point is retained; otherwise, the point is deleted, and the specific equation is shown in (17)^27^.

17 $$ D_{P} = \left\{ {\begin{array}{*{20}l} {D_{bP} \, if|D_{bq} - D_{mq} | \le 1} \hfill \\ {D_{invalid} \, otherwise} \hfill \\ \end{array} } \right. $$

**Proper interpolation:** Different interpolation methods are used to fill in mismatched pixels and pixels in occluded areas. For the pixels in the occluded area, since the occlusion point is very likely to come from the background, we select the pixel with the smallest disparity value for interpolation; otherwise, select the pixel with the closest color for interpolation. For mismatched pixels, we look for the closest reliable pixel in 8 different directions.

**Sub-pixel enhancement:** A sub-pixel enhancement method based on quadratic polynomial interpolation is adopted to reduce the error caused by discrete disparity levels^28^. For each pixel $p$, the optimal subpixel disparity is calculated by Eq. (18). ^25^:

18 $$ d_{sub} = d - \frac{{C^{\prime}(p,d_{ + } ) - C^{\prime}(p,d_{ - } )}}{{2(C^{\prime}(p,d_{ + } ) + C^{\prime}(p,d_{ - } ) - 2C^{\prime}(p,d))}} $$

where $d$ is the disparity depth with the least cost, $d_{ + } = d + 1$, $d_{ - } = d - 1$.

Using the left–right consistency detection, the disparity of the occluded area in the disparity map can be obtained, and then the background filling method is used to fill it, and then the sub-pixel enhancement method is used to improve the overall accuracy of the stereo matching algorithm, and finally, the disparity map is processed by median filtering. Smoothing to get the final optimized disparity map.

## Volume computation

Based on the disparity map obtained by stereo matching. By substituting the camera parameters obtained from camera calibration into Eqs. (7) or (8), the three-dimensional point coordinates of the measured object can be solved, and then the point cloud data of the measured object can be obtained. There will be noise in the initially obtained point cloud, which is first processed using a filtering method. After that, the point cloud is meshed using the Delaunay triangulation method, and then the volume of the target object is calculated by the projection method. The projection method is to divide the point cloud into several combinations whose volumes can be obtained, obtain the volume of each combination, and sum the volumes of all the combinations to obtain the overall volume of the measured object.

### Point cloud filtering

In the process of calculating the three-dimensional point coordinates of the object surface, affected by the errors in the steps of camera calibration, image correction and stereo matching, noise points and distortion points will inevitably be generated in the initially obtained point cloud data. Generally, the filtering method is used to denoise the point cloud to make the point cloud smooth. Fleishman^29^ applies it to 3D point cloud data on the basis of image bilateral filtering, and its definition is shown in Eq. (19). Bilateral filtering is a nonlinear non-iterative filtering method that uses the weighted average of the gray values of the pixels in the neighborhood to replace the gray value of the current point. This method can smooth not only the point cloud but also protect the edge features of the point cloud data.

19 $$ P_{i} ^{\prime} = P_{i} + \alpha \overrightarrow {{n_{i} }} $$

where, $P_{i}$ is the original point cloud, $P_{i} ^{\prime}$ is the point cloud after denoising, $\overrightarrow {{n_{i} }}$ is the normal vector of $P_{i}$ points, and 5 is the weight factor of bilateral filtering, and its definition is shown in Eq. (24). ^30^:

20 $$ \alpha = \frac{{\sum {_{{P_{j} \in K(P_{i} )}} W_{c} \left( {\left\| {P_{j} - P_{i} } \right\|} \right)W_{s} \left( {\left\| {\left\langle {\overrightarrow {{n_{i} }} ,P_{j} - P_{i} } \right\rangle } \right\|} \right) \cdot \left\langle {\overrightarrow {{n_{i} }} ,P_{j} - P_{i} } \right\rangle } }}{{\sum {_{{P_{j} \in K(P_{i} )}} W_{c} \left( {\left\| {P_{j} - P_{i} } \right\|} \right)W_{s} \left( {\left\| {\left\langle {\overrightarrow {{n_{i} }} ,P_{j} - P_{i} } \right\rangle } \right\|} \right)} }} $$

where $K(P_{i} )$ represents the neighborhood point of the point cloud $P_{i}$, $\left\| * \right\|$ represents the modulus of the vector, 〈〉 represents the inner product of the vector, $W_{s}$ represents the feature preservation weight function, and $W_{c}$ represents the smoothing filter weight function.

21 $$ W_{c} = e^{{ - x^{2} /(2\sigma_{c}^{2} )}} $$

22 $$ W_{s} = e^{{ - x^{2} /(2\sigma_{s}^{2} )}} $$

where $\sigma_{c}$ represents the smoothness of the point cloud and $\sigma_{s}$ represents the feature retention characteristics of the point cloud.

### Delaunay triangulation algorithm

Delaunay triangulation results have good triangulation properties so that ill-conditioned triangles do not appear in the triangulation results. And the triangles divided by Delaunay are unique and do not overlap each other, and the circumcircle of each triangle does not contain other vertices. If the diagonals of a convex quadrilateral formed by any two adjacent triangles can be interchanged, the smallest of the six interior angles of the two triangles will not become larger. The triangular shape can maximize the minimum angle so that each triangle is as close to an equilateral triangle as possible. Adding, deleting, or moving a vertex will only affect the adjacent triangles.

**Figure 3:**
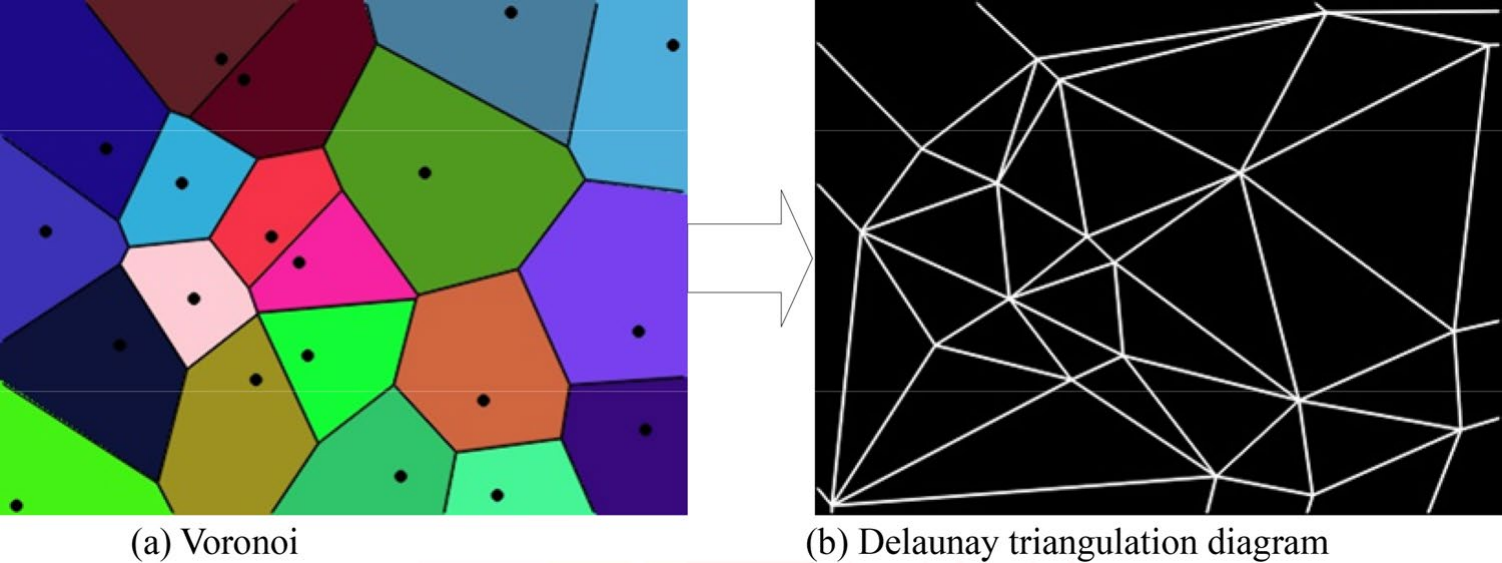
Voronoi and delaunay triangulation.

Inseparable from the Delaunay triangulation is the Voronoi^31^. Taking a two-dimensional plane point set as an example, first, draw a vertical bisector between every two points, and then connect them to each other so that the final topological structure is a Voronoi diagram, as shown in Fig. 3a. The Voronoi region is a regional structure of the Voronoi diagram. For a point set $S = \left\{ {p_{1} ,p_{2} , \cdots ,p_{n} } \right\}$ in two-dimensional space, for any point $p_{i}$ in it, the Voronoi region where it is located can be expressed as:

23 $$ V(p_{i} ) = \left\{ {p \in R^{2} \left| {\delta (p,p_{i} )} \right. < \delta (p,p_{j} ),j = 1,2, \cdots i - 1,i + 1, \cdots ,n} \right\} $$

where $\delta (p,p_{i} )$ represents the distance between point *p* and point $p_{i}$.

If the concept of the Voronoi diagram is extended to three-dimensional space, the Voronoi region will no longer be divided by straight line segments but by the nearest neighbor made by the vertical bisector of two points. At this time, the Voronoi area is no longer a polygonal area but a polyhedral area. In fact, the Voronoi diagram and Delaunay triangulation have been proved to be dual: in the Voronoi diagram, the triangulation topology is obtained by connecting the corresponding vertices of all adjacent Voronoi regions is the Delaunay triangulation, as shown in Fig. 3b.

### Object volume computation

**Figure 4:**
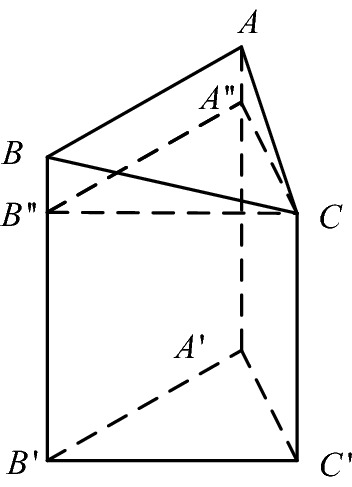
Triangular patch composite model.

After the point cloud of the measured object is obtained, the point cloud is first projected onto the plane. At this time, the projected points are scattered points in the two-dimensional plane, and the projected scattered points are subjected to Delaunay triangulation. According to the division standard of Delaunay triangulation, it can be seen that the triangular triangles are adjacent and do not contain each other, and the vertex of each triangulation triangle corresponds to a projection point in the bulk point cloud model. Then each triangulation triangle has a one-to-one correspondence with a triangular patch on the surface of the bulk, and the projected triangle and the triangular patch can form a combination. As shown in Fig. 4. It can be known that each combination is composed of two tetrahedrons and a triangular prism, and the volume $V_{i}$ of each combination is equal to the sum of the volumes of these three spatial bodies.

24 $$ V_{i} = V_{ABC - A^{\prime}B^{\prime}C^{\prime}} + V_{B - B^{\prime\prime}A^{\prime\prime}C} + V_{A - BA^{\prime\prime}C} $$

It is assumed that the projected point cloud is divided into M triangles after Delaunay triangulation. It can be known that the entire stacking object can be divided into M composite bodies. As shown in Fig. 4, the volume of each composite body is calculated, and the volume of all composite bodies is accumulated to obtain the volume V of the stacked body.

25 $$ V{ = }\sum\limits_{i = 1}^{M} {V_{i} } $$

When the number of point clouds obtained is more, the description of the surface features of the stacked objects is complete. After the projected point cloud is triangulated, the more triangles and combinations are obtained, and the volume obtained in this way, the more accurate the result.

## Experimental results and analysis

The parameters of the two cameras need to be known when calculating the coordinates of the space point by Eqs. (7) and (8), and the process of obtaining the camera parameters (including the camera internal parameter matrix and the camera external parameter matrix) is called camera calibration. The accuracy and reliability of the calibration results will affect the accuracy and stability of stereo matching. Since the Zhang Zhengyou calibration method^32^ has the advantages of simple operation and accurate calibration results, the Zhang Zhengyou calibration method is used to calibrate the binocular camera, and the obtained parameters are shown in Table 1:

**Table 1:** Camera parameters.

Name	Value
Left camera intrinsic parameter matrix	$$\left[ {\begin{array}{*{20}c} {807.35} & 0 & {363.86} \\ 0 & {806.75} & {240.07} \\ 0 & 0 & 1 \\ \end{array} } \right]$$
Left camera distortion factor	$$\left[ {\begin{array}{*{20}c} { - 0.0192} & {0.1908} & 0 & 0 & 0 \\ \end{array} } \right]$$
Right camera intrinsic parameter matrix	$$\left[ {\begin{array}{*{20}c} {806.31} & 0 & {337.45} \\ 0 & {805.62} & {235.51} \\ 0 & 0 & 1 \\ \end{array} } \right]$$
Right camera distortion factor	$$\left[ {\begin{array}{*{20}c} { - 0.0122} & {0.0588} & 0 & 0 & 0 \\ \end{array} } \right]$$
Rotation matrix	$$\left[ {\begin{array}{*{20}c} {0.9999} & { - 0.0008} & { - 0.0083} \\ {0.0009} & {0.9999} & {0.0063} \\ {0.0083} & { - 0.0006} & {0.9999} \\ \end{array} } \right]$$
Translation vector	$$\left[ {\begin{array}{*{20}c} { - 61.6635} & { - 0.1372} & { - 1.5766} \\ \end{array} } \right]$$

**Figure 5:**
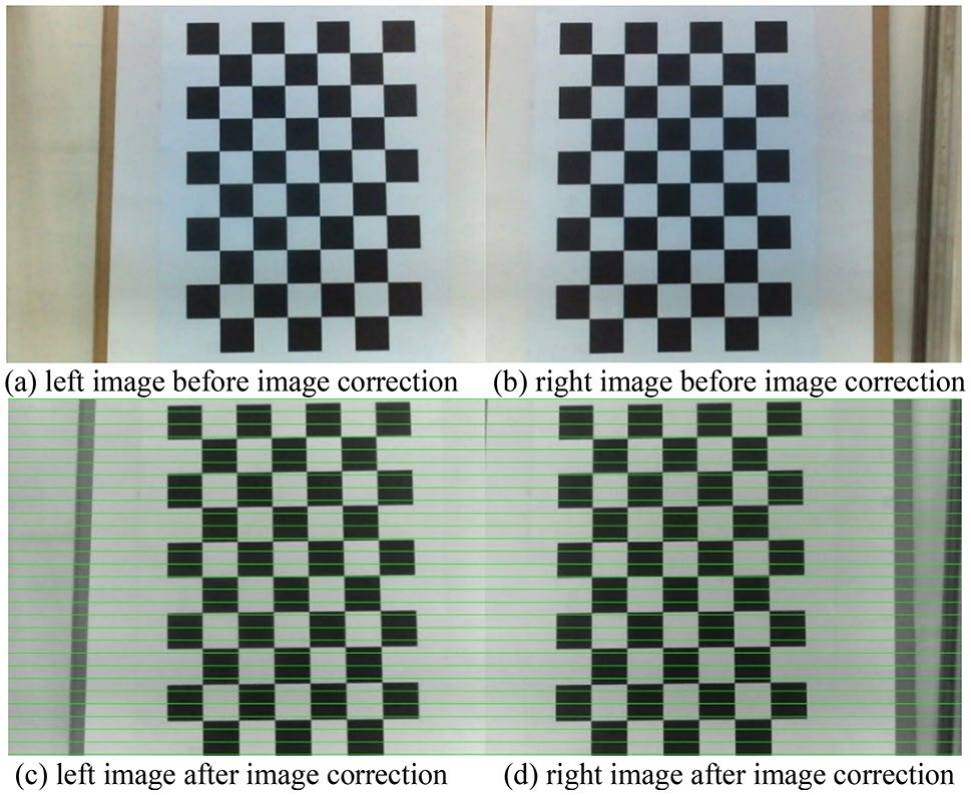
Image correction processing.

Since the input image pair for stereo matching is an image that has been corrected, the image needs to be corrected before performing stereo matching. The purpose of image correction is to make the corresponding epipolar lines of the two images lie on the same scan line and parallel to the camera baseline. At the same time, the search range of stereo matching can be reduced from two-dimensional to one-dimensional^41^. When searching for the corresponding point of the two images, it is only necessary to search on the scanning line corresponding to the point so that the stereo matching problem can be simplified and effectively dealt with. We use the Bouguet method^33^ for image correction, which is image correction using the intrinsic parameter matrix obtained from camera calibration. The processed images before and after image correction are shown in Fig. 5:

The stereo matching experiment uses the dataset provided by the Middlebury^11^ evaluation platform to verify the stereo matching algorithm. Use $d_{t} (p)$ for the true disparity value, $d_{r} (p)$ for the calculated disparity value, and if $\left| {d_{t} (p) - d_{r} (p)} \right| > 1$, then $d_{r} (p)$ is considered a false disparity value. In the specified area, the total number of error values is divided by the total number of valid points (excluding the points with no true disparity value) to obtain the error rate of $R$, which is used as an index to evaluate the accuracy and performance of the algorithm.

### Analysis of stereo matching parameters

This set of experiments is to analyze the effect of the magnitude of the $\lambda_{C}$, $\lambda_{SD}$ and $\lambda_{G}$ parameter values on the generation of disparity maps. First, setting $\lambda_{C}$ = 30, $\lambda_{SD}$ = 30, change the value of $\lambda_{G}$ and calculate the disparity error matching rate corresponding to each $\lambda_{G}$, and determine the value of $\lambda_{G}$ by the size of the error matching rate. Then, setting $\lambda_{SD}$ = 30, change the value of $\lambda_{C}$ and calculate the disparity error matching rate corresponding to each $\lambda_{C}$, and determine the value of $\lambda_{C}$ by the size of the error matching rate. Finally, determine the value of $\lambda_{SD}$.

**Figure 6:**
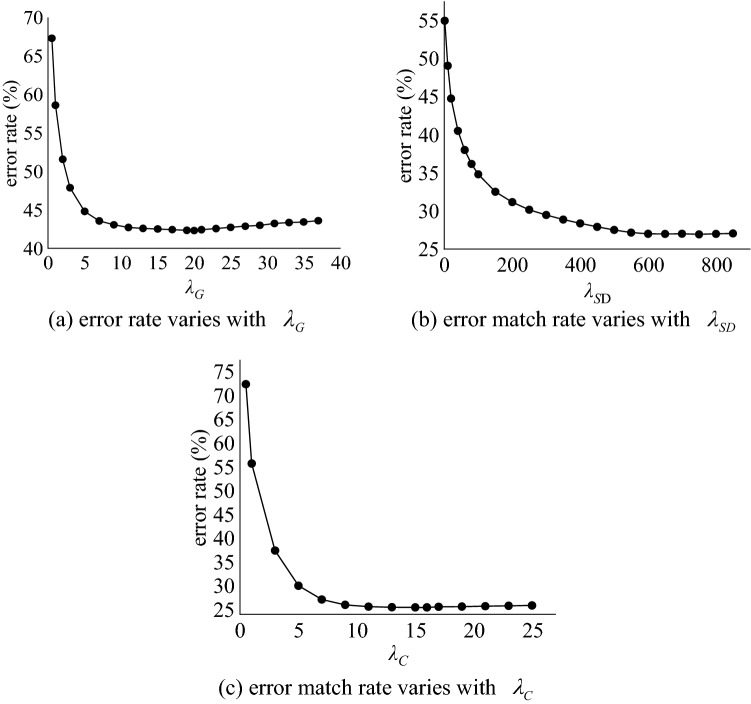
The effect of parameter changes on the error rate.

**Figure 7:**
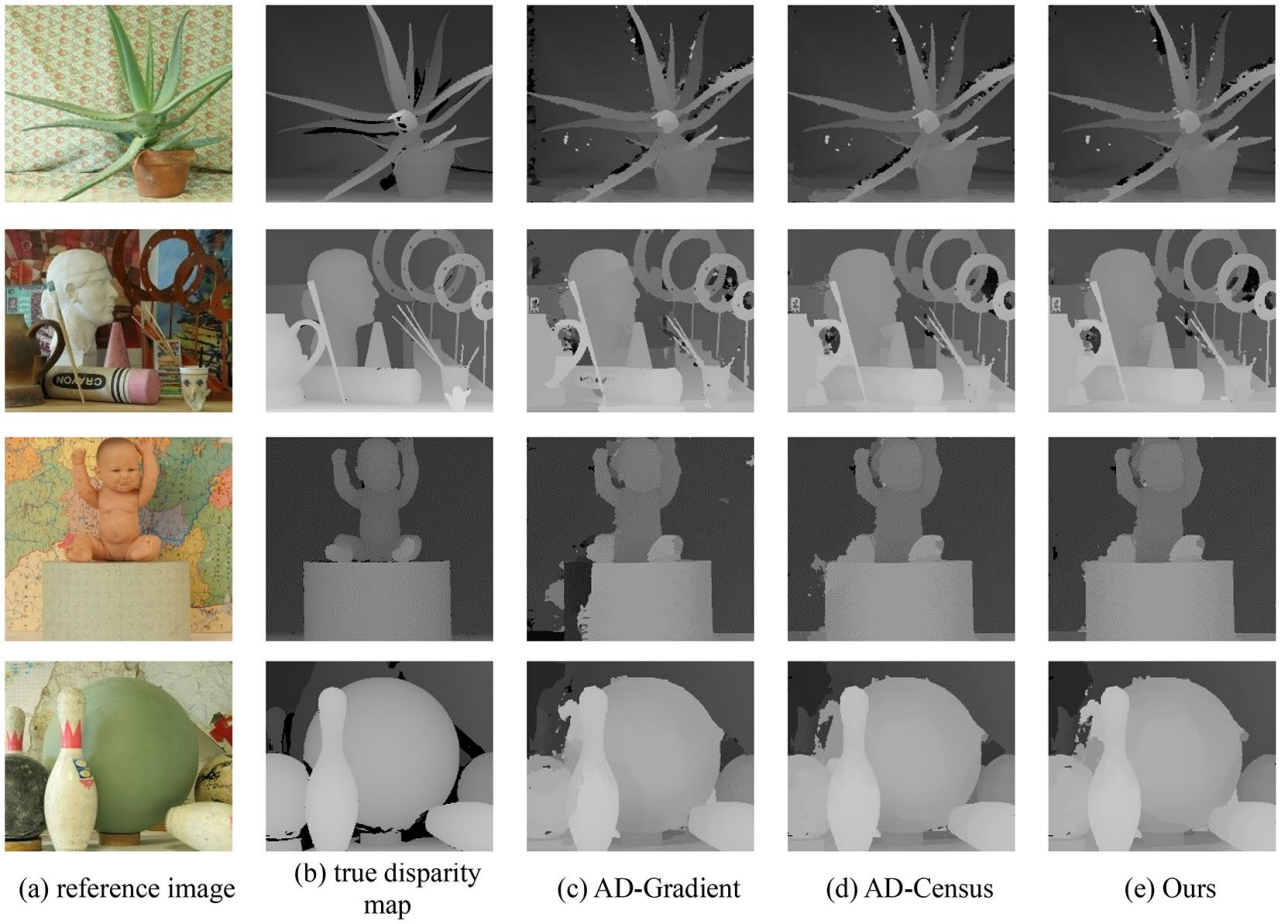
Disparity maps generated by different cost computation methods.

In order to clearly observe the changing laws of different parameter values on different images, we selected six groups of images for experiments and calculated the mean value of the error rate. The results are shown in Fig. 7. Figure 6a shows the change rule of $\lambda_{G}$. It can be seen from the figure that when $\lambda_{G}$ is equal to 20, the error rate is the lowest. Figure 6b shows the change rule of $\lambda_{SD}$. It can be seen from the figure that the error rate of $\lambda_{SD}$ fluctuates less after 600. Figure 6c shows the change rule of $\lambda_{C}$. It can be seen from the figure that when $\lambda_{C}$ is between 13 and 21, the error rate is relatively flat. When it is equal to 15, the error rate is the lowest, and after 21, the error rate fluctuates. There is an upward trend.

The rest of the parameters use the experimental parameters in the original reference, and the experimental parameters are set as shown in Table 2:

**Table 2:** The specific parameter values of the experiment.

Parameter	$\lambda_{C}$	$\lambda_{SD}$	$\lambda_{G}$	$L_{1}$	$L_{2}$	$\tau_{1}$	$\tau_{2}$	$\Pi_{1}$	$\Pi_{2}$	$\tau_{SO}$	$\tau_{S}$	$\tau_{H}$
Value	15	600	20	34	17	20	6	1.0	3.0	15	20	0.4

### Verification of matching cost computation method

In order to verify the effectiveness of the matching cost calculation method proposed in this paper, we choose AD-Gradient^34^ and AD-Census^25^as comparative experiments. Experiments are performed using 12 sets of stereo image pairs, and the mismatch rates of all regions are calculated, and the results are shown in Table 3. As can be seen from the table, our matching cost computation method is better than AD-Gradient and AD-Census cost computation methods.

**Table 3:** Mismatching rate of all pixel disparity for different cost computation methods(%).

Method	Aloe	Baby1	Books	Art	Bowling1	Cloth1
AD-gradient	15.619	30.249	39.199	43.969	40.329	31.319
AD-census	11.429	19.949	33.109	38.479	33.559	24.149
Gradient-SD-census	10.439	18.129	30.089	36.689	31.639	16.039

Method	Cloth3	Baby2	Dolls	Cloth2	Bowling2	Lampshade1
AD-gradient	22.589	38.019	40.269	41.859	49.539	45.249
AD-census	16.159	24.989	28.659	33.979	37.899	38.427
Gradient-SD-census	15.759	20.729	26.389	32.829	36.249	32.178

Figure 7 shows the disparity images generated by the three matching cost computation methods in the four images of Aloe, Art, Baby1, and Bowling2. From the figure, it can be observed that our algorithm outperforms other algorithms in edge regions and weakly textured regions. And the reasons why the algorithm is effective are analyzed. Both Census Transformation and gradient describe the local structure. The former describes a large range, while the latter describes a small range. In the weak texture area, the color difference of the pixel points is very small (the points in the untextured area also have a color difference, but the difference is smaller), and only using the SD of a single point to calculate the cost will produce very large ambiguity. This is because there are many similarities within the parallax range. When the disparity of most points in the aggregation area is ambiguous, the cost value after aggregation will be very unreliable. Census Transform can use the small color difference between pixels to encode the local structure of the target point, and the ambiguity of the local structure is much smaller than the intensity of a single point, so the Census Transform cost has a natural advantage for weak textures. The local structure information in the gradient is contained in the intensity changes between the left and right adjacent points and the upper and lower adjacent points of the target point. This smaller local structure is still effective for the characterization of weak texture areas. In summary, our algorithm obtains a matching cost that is robust to weak texture regions by combining gradient cost, SD, and Census Transform.

### Overall performance analysis of stereo matching algorithm

**Figure 8:**
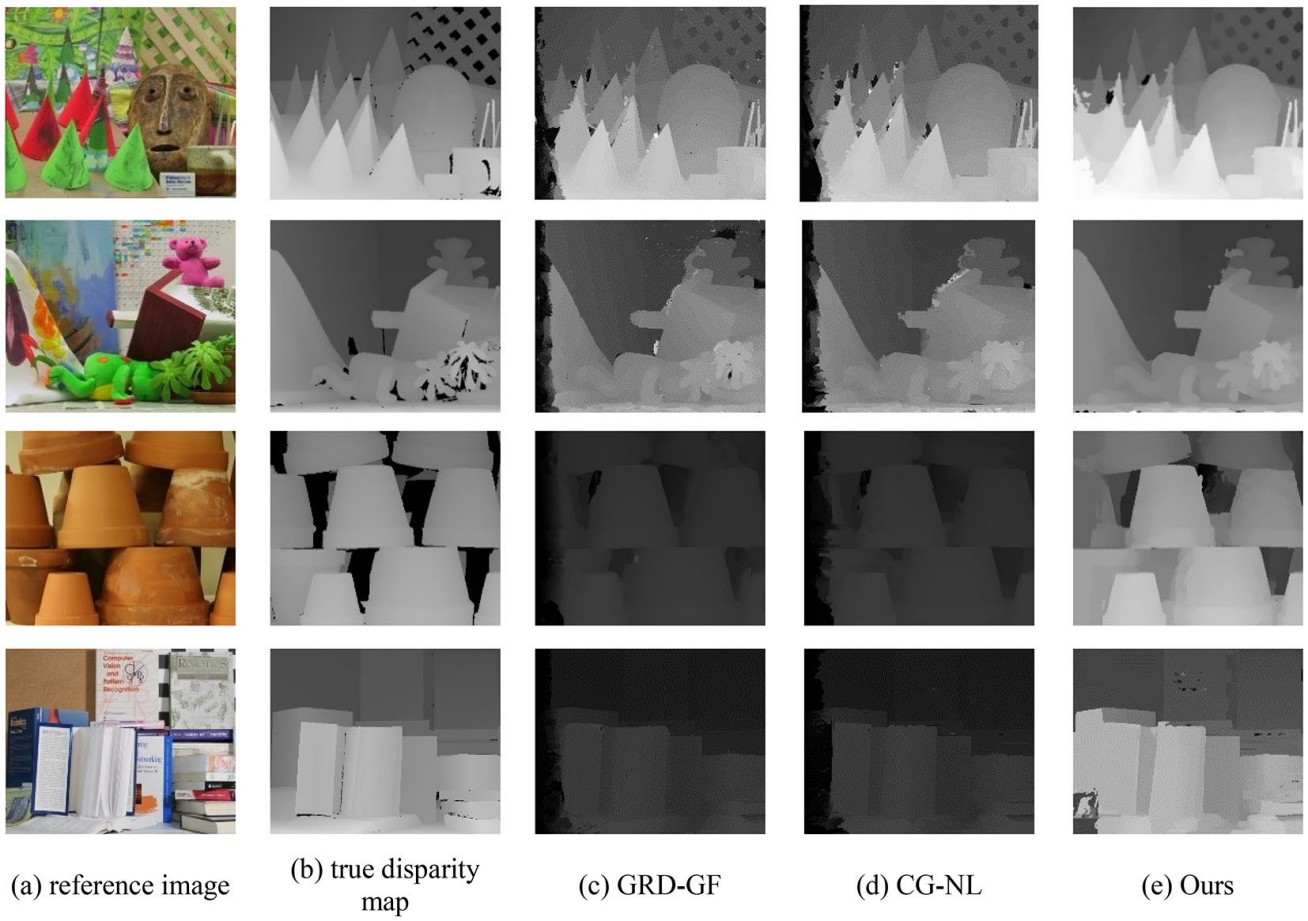
Disparity maps generated by different algorithms.

In order to objectively evaluate the overall evaluation of the algorithm in this paper, 12 pairs of stereo matching images in Middlebury were selected to test the algorithm. And compare the disparity map of the algorithm in this paper with the Truncated Threshold Absolute Difference + Gradient + guided filter cost aggregation algorithm (GRD-GF)^35,36^ and Census + gradient + non-local cost aggregation algorithm (CG-NL)^37^. The guided filter uses the image itself or another different image as the input image to calculate the filtering output, which has the functions of protecting the edge and denoising and can improve the matching accuracy at the edge of the image. The non-local cost is aggregated on the tree structure of the stereo image, and the matching cost value is adaptively aggregated based on pixel similarity to preserve the depth edge. Table 4 is a comparison of the disparity average error of all pixels of different stereo matching algorithms. Figure 8 shows the true disparity map and the disparity map generated by different algorithms.

**Table 4:** Mismatching rate of all pixel disparity for different stereo matching algorithms(%).

Method	Aloe	Baby1	Books	Cones	Bowling1	Cloth1
GRD-GF	19.428	20.412	33.412	41.169	35.149	18.246
CG-NL	17.521	21.458	32.154	41.025	33.059	19.419
Ours	10.439	18.129	30.089	36.689	31.639	16.039

Method	Cloth3	Baby2	Dolls	Teddy	Bowling2	Lampshade1
GRD-GF	17.658	24.582	30.028	41.318	39.527	38.151
CG-NL	17.255	26.412	29.514	40.159	38.949	36.149
Ours	15.759	20.729	26.389	37.728	36.249	32.178

### Experiment results and analysis of point cloud volume computation

Due to the irregular shape, size, and surface of experimental mice feed, accurate volume measurements are difficult. When using the feed for experiments, the accumulation and collision of the feed will cause some particles on the surface of the feed to fall off, resulting in changes in the actual volume of the feed, which is difficult to apply to repeated experiments. The measurement of the actual volume of the experimental mice feed is the value obtained by data processing on the basis of repeated experiments. Therefore, a cylindrical wooden stick with a smooth surface and a denser material was used to replace the actual feed, and repeated measurement experiments were carried out. Therefore, different specifications of wood are used instead of the mice feed in the volume calculation experiment. The specifications of wood include diameters of 10 mm, 12 mm, and 15 mm, heights of 20 mm and 30 mm, and a number of them.

**Figure 9:**
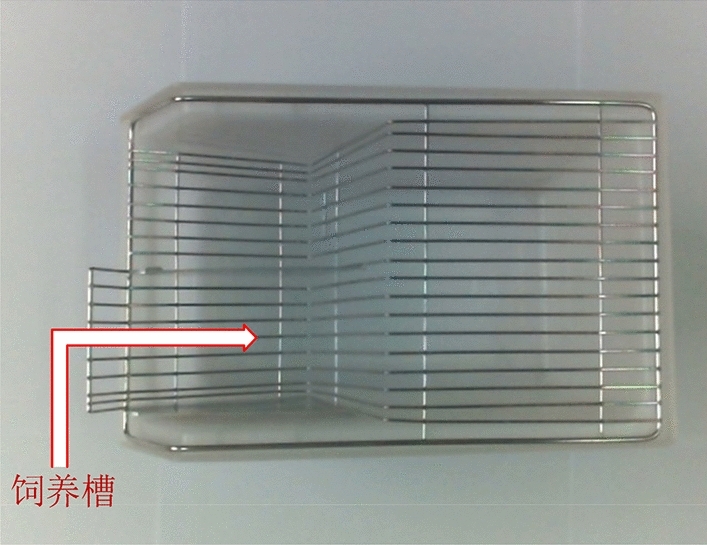
Original feeding box image.

**Figure 10:**
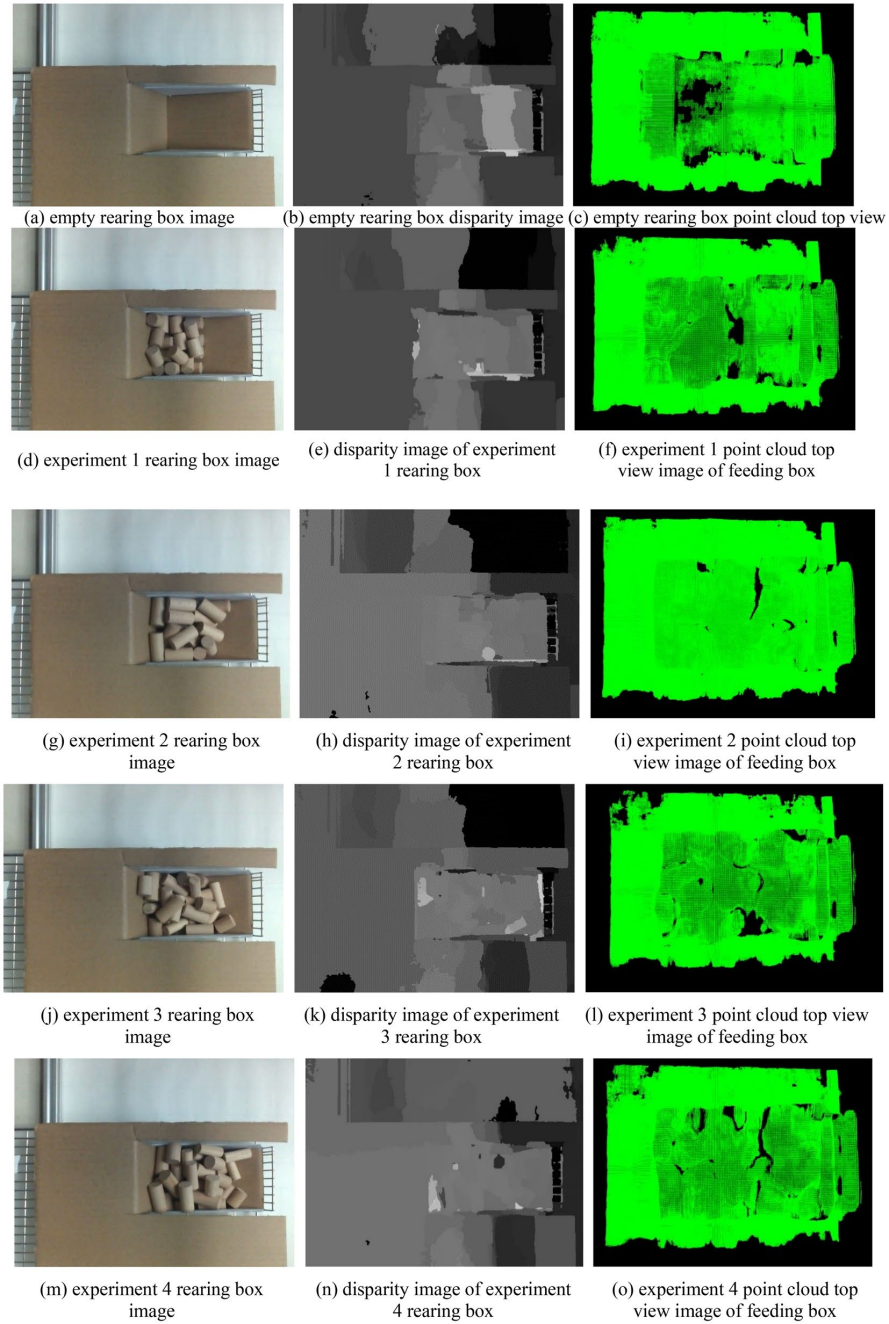
The feeding box image, stereo matching result, and point cloud image.

Due to the particularity of the mice rearing box, it is first necessary to perform stereo matching on the empty rearing box to obtain the three-dimensional point coordinates of its surface. Then, stereo matching is performed again on the feeding box containing the feed, and the three-dimensional point coordinates of its surface are obtained. The three-dimensional point coordinates obtained twice are subtracted, and the points with the same coordinates are eliminated, and then the point cloud data of the feed is obtained. The position of the camera and the rearing box remained unchanged during this process. The original image of the mice rearing box is shown in Fig. 9, and the mice's food is placed in the rearing tank shown in the figure. There is a certain angle between the feeding trough and the horizontal plane, and the feeding trough is hollow, which will seriously affect the result of stereo matching. Therefore, the rearing box is packaged with cardboard, as shown in Fig. 10a:

The next step is to perform stereo matching between the empty rearing box and the rearing box with feed and obtain the point cloud data of the entire rearing box. The results are shown in Fig. 10.

**Figure 11:**
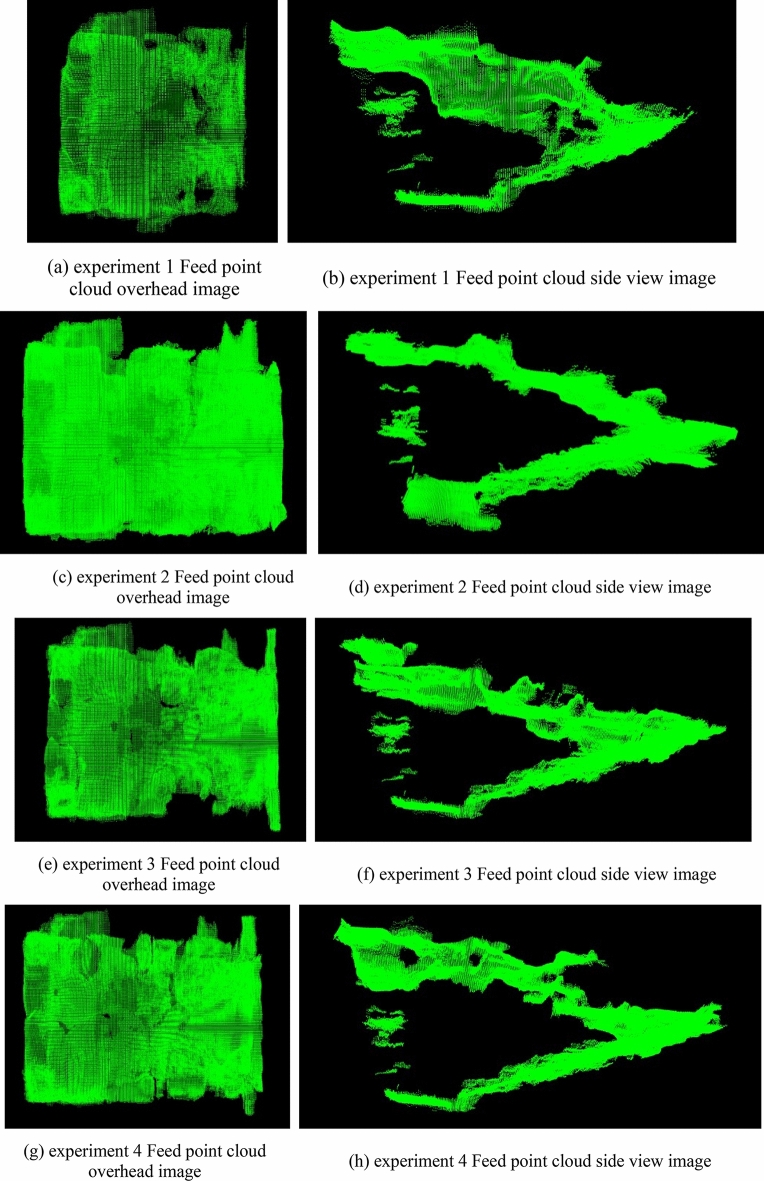
Point cloud image.

After obtaining the point cloud of the empty rearing box and the rearing box with the feed, the subtraction operation is performed to obtain the point cloud of the feed alone, as shown in Fig. 11:

Experiment 1, experiment 2, experiment 3, and experiment 4 in Figs. 10 and 11 correspond to experiment numbers 1, 2, 3, and 4. According to the volume calculation principle in “Volume computation”, the volume of the mice feed was calculated, and the results are shown in Table 5.

**Table 5:** Data analysis of experimental results.

Experiment number	Actual volume (mm^3^)	Measure volume (mm^3^)	Volume error rate (%)
1	88,357.25	93,948.52	6.33
2	121,933.12	130,420.14	6.96
3	122,286.50	131,097.15	7.20
4	145,848.40	156,241.75	7.13
5	156,215.65	168,347.78	7.76
6	158,972.45	169,986.95	6.93
7	173,887.20	184,535.49	6.12
8	176,714.60	187,965.68	6.37
9	175,300.80	188,946.27	7.78
10	91,891.60	98,768.57	7.48
11	136,580.70	147,253.26	7.81
12	91,341.80	98,251.46	7.56
Average	136,610.80	146,313.59	7.12

It can be seen from the table that the volume error rate of each test is below 8%, and the average error is 7.12%. The data in the table can reflect the feasibility of using this method to calculate the volume of mice feed. Since the obtained point cloud is the point cloud on the surface of the feed, gaps will inevitably appear in the feed when the mice feed is piled up. The existence of these gaps expands the real space of the feed. As a result, the calculated volume is higher than the real volume every time, which in turn has a certain impact on the accuracy of the volume calculation.

## Conclusion

Aiming at the problem of volume calculation in the way of food bulk in mice, this paper proposes a method for detecting the bulk volume of food in mice based on binocular vision. Firstly, the binocular stereo vision three-dimensional reconstruction technique is used to calculate the three-dimensional point coordinates of the feed surface, and then the coordinates of a series of dense points are obtained to form a point cloud. And then, the volume of the point cloud is calculated by the projection method to obtain the volume of the mice feed. Ther are aiming at the problem that the stereo matching algorithm in the binocular vision three-dimensional reconstruction technology has a high mismatch rate in weak texture regions. In the matching cost computation stage, the image gradient information, SD, and Census Transform are weighted and fused to obtain the initial matching cost and the Cross-Based Cost Aggregation. Then, the initial disparity is obtained through the WTA rule, and the disparity map is optimized using strategies such as left–right consistency detection and sub-pixel refinement. Finally, the three-dimensional point coordinates of the feed are calculated through the principle of triangulation. Experiments are carried out using the stereo matching image dataset provided by the Middlebury evaluation platform to verify the effectiveness of the proposed initial matching cost computation method. After obtaining the point cloud of the feed, first, use bilateral filtering to denoise the point cloud, then use the Delaunay triangulation algorithm to tetrahedral the point cloud, and finally accumulate and sum each tetrahedron to obtain the total volume of the feed. We use wood of different sizes instead of feed for volume calculation, and the average error between the calculated volume and the real volume after many experiments is 7.12%. The experimental results show that the volume of the remaining feed of mice can be calculated by binocular vision.

The way the mice feed is accumulated will inevitably generate gaps, and the size of the gaps is also uncontrollable. The gaps are an important factor for errors in the volume calculation process. The idea of reducing the error caused by this factor is as follows: put the same amount of feed in the feeding tank in different ways and then calculate the corresponding volume each time. After obtaining multiple sets of data, perform data fitting on them to obtain a suitable one. A function is used to express the relationship between the calculated volume and the real volume so as to obtain higher calculation accuracy.

## Data Availability

Data is contained within the article or supplementary material. The data presented in this study are available in this article.
